# Transcriptomic signature associated with RNA-binding proteins for survival stratification of laryngeal cancer

**DOI:** 10.18632/aging.204234

**Published:** 2022-08-18

**Authors:** Yujie Shen, Huijun Chen, Qiang Huang, Huaidong Du, Liang Zhou

**Affiliations:** 1 Department of Otorhinolaryngology Head and Neck Surgery, Eye, Ear, Nose, and Throat Hospital, Fudan University, Shanghai 200031, Shanghai, China; 2 Department of Otolaryngology Head and Neck Surgery, Zhongda Hospital, Southeast University, Nanjing 210009, Jiangsu, China

**Keywords:** RNA-binding proteins, laryngeal squamous cell carcinoma, overall survival, prognosis, signature

## Abstract

RNA-binding proteins (RBPs) have been suggested as important prognostic indicators in different human cancers. This study was designed to search the prognostic value of RBPs of laryngeal squamous cell carcinoma (LSCC). Differentially expressed RBPs (DERBPs) were screened via The Cancer Genome Atlas (TCGA). Bioinformatics methods were used to identify prognostic DERBPs. Expression profiling of training cohort were calculated to develop a transcriptomic signature, which was validated by three independent cohorts (TCGA cohort, GSE65858 cohort and GSE27020 cohort). We identified DERBPs and a set of signatures (GTPBP3, KHDRBS3 and RBM38) were confirmed as prognosis-related hub DERBPs in LSCC, which was also tested and verified by bioinformatics method and molecular biology experiment. The role of immune cell infiltration and drug resistance between subgroups was explored. Furthermore, the risk score based on transcriptomic signature was turned out to be an independent prognostic indicator for LSCC. Finally, a nomogram for further clinical application was established. Our study demonstrated that the transcriptomic signature we constructed could serve as a novel therapeutic target and biomarker for LSCC from the perspective of RBPs.

## INTRODUCTION

Laryngeal squamous cell carcinoma (LSCC), a frequent and invasive malignancy of head and neck squamous cell carcinoma (HNSCC), is the eighth largest cause of cancer-associated death in the world [1]. The morbidity of LSCC is higher in men than in women and the leading pathogenic risk factors includes smoking and alcohol drinking [2]. As the proportion of people over 70 years of age rises in the past twenty years, the incidence of LSCC is growing globally and the burden may continue to ascend in the future [3, 4]. Conventional management approaches to laryngeal cancer include radical surgery, radiation therapy, and chemotherapy. However, to date, there is still a poor prognosis for laryngeal cancer.

RNA-binding proteins (RBPs) [5] participate in many biological activities, such as the modulation of pre–messenger RNA (mRNA) splicing and RNA modification, translation, stability, and localization [6]. RBPs identify targets and regulate co-transcriptional RNA, post-transcriptional processes by RNA-binding domains [7]. Thus far, some RBPs have been identified in human cell lines and tumors through genome-wide analysis [8]. Statistically, approximately half of these RBPs can be grouped according to their mRNA targets, while others can interact with different RNA types. Moreover, about one third of the RBPs can combine with DNA and RNA [9]. The abnormal expression of RBPs has been detected in multiple human tumor types, and different RBPs can control different processes in RNA metabolism of the target genes [10]. For example, RNA-binding motif protein 47 (RBM47) can suppress progression and metastasis in breast cancer cells [11].

RBPs are known to be linked to the development, and metastasis of urothelial carcinoma of bladder [12]. MSI1, a RBP of the Musashi family, is associated with oral squamous cell carcinoma [13], gastric carcinoma [14], and carcinoma of the lungs [15]. However, the prognostic value of RBPs for laryngeal cancer is rarely reported so far. In our study, we intended to establish and verify a novel prognostic signature in view of RBPs expression profile for LSCC patients by integrating different independent cohorts.

## MATERIALS AND METHODS

### Detection of DERBPs

Transcript data were obtained from TCGA (https://portal.gdc.cancer.gov), which containing 111 LSCC samples and 12 paired samples. In Supplementary Table 1, detailed clinical information is presented. The RNA-seq data were processed by using “limma” package of the R software. | log (FC) | ≥ 0.5 and an adjusted *p*-value < .05 were defined as the screening criteria for detecting DERBPs between healthy laryngeal tissues and LSCC tissues from TCGA.

### Pathway enrichment analysis

To identify the predominant biological processes of the DERBPs, gene ontology (GO) [16] and Kyoto encyclopedia of Genes and Genomes (KEGG) pathway analysis [17] were performed. An adjusted *p*-value < .05 was considered as a screening criterion.

### PPI network establishment

To screen hub genes that were tightly linked to each other, DERBPs were introduced into the String database (https://string-db.org/) and Cytoscape software (version: 3.7.1). The plug-in unit “MODE” was used for sub-network construction. We then performed pathway enrichment and visualized the sub-networks.

### GEO cohorts

To increase the reliability of the prognostic signature, we integrated GSE27020 dataset (109 patients) from GEO database for DFS analysis. Thirty-four recurrent and seventy-five non-recurrent LSCC patients were collected as external cohorts for further validation. GSE27020 dataset was derived from the GPL96 platform (Affymetrix Human Genome U133A Array). Moreover, GSE65858 dataset including 270 HNSCC patients was further screened out and selected for validation of OS, which was derived from GPL10558 platform.

### Identification and validation of the transcriptomic signature

To develop a RBPs-related prognostic signature with good predictive performance, 111 LSCC patients were randomly assigned to training (56 patients) and test cohort (55 patients). The univariate Cox regression analysis was served to select the prognostic DERBPs in training cohort, and LASSO analysis method was performed to avoid overfitting. Multivariate Cox regression analysis was applied to determine the prognostic signature. The DERBPs with a *p*-value < .05 were considered as hub prognosis-related DERBPs. The calculation formula of the risk score based on training cohort was described below:

Risk score = coef A * A Expression + coef B * B Expression + coef i * i Expression [18–20]

On the basis of the median risk score, all samples were sorted into either the low- or high-risk group in training cohort. We compared the overall survival rate of the two groups by Kaplan-Meier methods, and evaluated the transcriptomic signature by plotting the receiver operating characteristic (ROC) curves for training, test, entire, GSE65858 and GSE27020 cohort.

### Immune infiltration and drug sensitivity analysis

In this study, cell-type identification by estimating relative subsets of RNA transcripts (CIBERSORT) algorithm [21] and single sample gene set enrichment analysis (ssGSEA) algorithm [22] were carried out on LSCC patients of TCGA cohort. The Wilcoxon test was applied to compare the immune cell infiltration of LSCC patients in different groups with the *p* value < .05 as statistically significant. Further, the RBPs expression of TCGA cohort were uploaded to Tumor Immune Dysfunction and Exclusion (TIDE) website (http://tide.dfci.harvard.edu/) in order to predict the immunotherapy response of the two different groups. Also, with the *p* value < .01 as statistically significant, effective chemotherapeutic drugs targeting low- or high-risk group were screened out via using Drug Sensitivity in Cancer (GDSC) database (https://www.cancerrxgene.org/) [23].

### Cell culture

Three LSCC cell lines and HuLa-PC were used to verify the gene expression. As described before [24], cell culture assays were performed. Moreover, a normal epithelial cell (HuLa-PC) was purchased from ATCC (Gaithersburg, MD, USA) and cultured in BEGM (CC-3170 Lonza).

### qRT-PCR

We performed real-time quantitative reverse transcriptase PCR (qRT-PCR) as described in our previous study [24]. The primers were synthesized by Sangon Biotech Co., Ltd. (Shanghai, China). Primer sequences are detailed in Supplementary Table 2.

### Independent prognosis analysis

Based on the entire cohort, we tried to judge whether the risk score that calculated by our prognostic signature was an independent prognostic factor for LSCC by univariate and multivariate Cox regression analyses.

### Nomogram construction

To better connect with the clinical application, we developed a nomogram by “rms” package of R software according to the result of independent prognosis analysis. Nomogram can assess the overall survival rate of patients with LSCC at different time points according to the scoring of independent prognostic factors. Time-dependent calibration curves were plotted to verify the effectiveness of nomogram internally.

### Statistical analysis

R software (version: x64 3.6.1) and GraphPad Prism7 were used for all statistical analyses in this study. The results of qRT-PCR were subjected to GraphPad Prism7 software by the Student’s t-test. A *p*-value < .05 was regarded as statistically significant.

## RESULTS

### Identification of DERBPs

**Figure 1:**
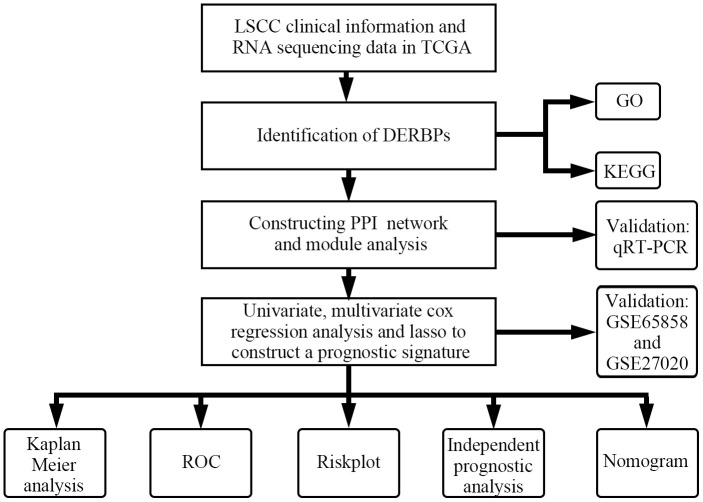
Flowchart of this study.

**Figure 2:**
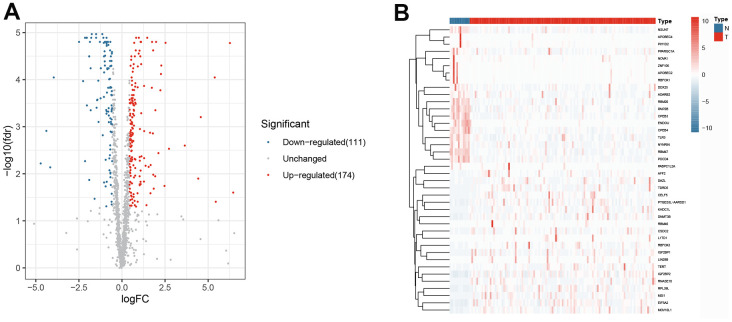
**The differentially expressed RNA-binding proteins from TCGA.** (**A**) Volcano plot; (**B**) Heat map.

The research design is illustrated in Figure 1. According to the screening criteria (adjusted *p*-value < .05, | log (FC) | ≥ 0.5), 285 DERBPs were identified (Supplementary Table 3). Among them, 111 down-regulated and 174 up-regulated DERBPs were identified (Figure 2).

### GO and KEGG analysis of DERBPs

The results indicated that down-regulated DERBPs were significantly enriched in the biological processes including mRNA processing, translation regulation, cellular amide regulation, metabolic processes, mRNA catabolic processes, and RNA catabolic processes (Table 1). The up-regulated DERBPs were significantly enriched in the non-coding RNA (ncRNA) metabolic processes, mRNA processing, RNA splicing, ncRNA processing, RNA splicing, and transesterification reactions (Table 1). According to the analysis of cellular component, down-regulated DERBPs were enriched in the ribosomal subunit, ribonucleoprotein granule, cytosolic ribosome, ribosome, and cytoplasmic ribonucleoprotein granule. On the other hand, the up-regulated DERBPs were mainly enriched in the spliceosomal complex, U2-type precatalytic spliceosome, precatalytic spliceosome, cytoplasmic ribonucleoprotein granule, and ribonucleoprotein granule (Table 1). According to the molecular function analysis, the down-regulated DERBPs were enriched during catalytic activity, acting on the RNA, AU-rich element binding, mRNA 3’-UTR AU-rich region binding, mRNA 3’-UTR binding, and ribonucleoprotein complex binding (Table 1), while the up-regulated DERBPs were significantly enriched during catalytic activity, acting on the RNA, nuclease activity, ribonuclease activity, transfer (tRNA), and tRNA binding (Table 1). Moreover, we found that down-regulated DERBPs were mainly enriched in mRNA surveillance pathway, ribosomes, RNA transport, and progesterone-mediated oocyte maturation; while up-regulated RBPs were significantly enriched in spliceosome, RNA transport, RNA degradation, mRNA surveillance pathway, and cytosolic DNA-sensing pathway (Table 1).

**Table 1:** Gene ontology pathway and Kyoto Encyclopedia of Genes and Genomes enrichment analysis of differentially expressed RNA-binding proteins.

	**Description**	***p* value**	***p*.adjust**
**Down-regulated RBPs**			
**BP**	mRNA processing	3.32E-17	4.98E-14
	regulation of translation	7.43E-13	5.58E-10
	regulation of cellular amide metabolic process	7.23E-12	3.62E-09
	mRNA catabolic process	6.79E-11	2.55E-08
	RNA catabolic process	2.38E-10	7.14E-08
**CC**	ribosomal subunit	2.89E-07	2.14E-05
	ribonucleoprotein granule	5.84E-07	2.14E-05
	cytosolic ribosome	6.23E-07	2.14E-05
	ribosome	7.51E-07	2.14E-05
	cytoplasmic ribonucleoprotein granule	3.75E-06	8.56E-05
**MF**	catalytic activity, acting on RNA	1.15E-11	2.39E-09
	AU-rich element binding	8.77E-09	4.78E-07
	mRNA 3'-UTR AU-rich region binding	8.77E-09	4.78E-07
	mRNA 3'-UTR binding	9.19E-09	4.78E-07
	ribonucleoprotein complex binding	2.40E-08	1.00E-06
**KEGG**	mRNA surveillance pathway	0.000158718	0.007591248
	Ribosome	0.00025733	0.007591248
	RNA transport	0.000518043	0.010188172
	Progesterone-mediated oocyte maturation	0.002382425	0.03514077
**Up-regulated RBPs**			
**BP**	ncRNA metabolic process	2.31E-32	3.98E-29
	mRNA processing	1.08E-26	8.80E-24
	RNA splicing	1.96E-26	8.80E-24
	ncRNA processing	2.04E-26	8.80E-24
	RNA splicing, via transesterification reactions	1.24E-20	4.26E-18
**CC**	spliceosomal complex	1.30E-10	2.26E-08
	U2-type precatalytic spliceosome	2.95E-10	2.26E-08
	precatalytic spliceosome	2.95E-10	2.26E-08
	cytoplasmic ribonucleoprotein granule	4.61E-10	2.65E-08
	ribonucleoprotein granule	1.10E-09	4.33E-08
**MF**	catalytic activity, acting on RNA	1.01E-30	2.15E-28
	nuclease activity	7.84E-14	8.35E-12
	ribonuclease activity	4.07E-13	2.89E-11
	catalytic activity, acting on a tRNA	8.50E-13	4.52E-11
	tRNA binding	2.46E-11	1.05E-09
**KEGG**	Spliceosome	1.19E-15	6.33E-14
	RNA transport	1.04E-08	2.75E-07
	RNA degradation	4.52E-08	7.99E-07
	mRNA surveillance pathway	2.20E-05	0.000291209
	Cytosolic DNA-sensing pathway	0.002889905	0.026712079

### PPI network construction

**Figure 3:**
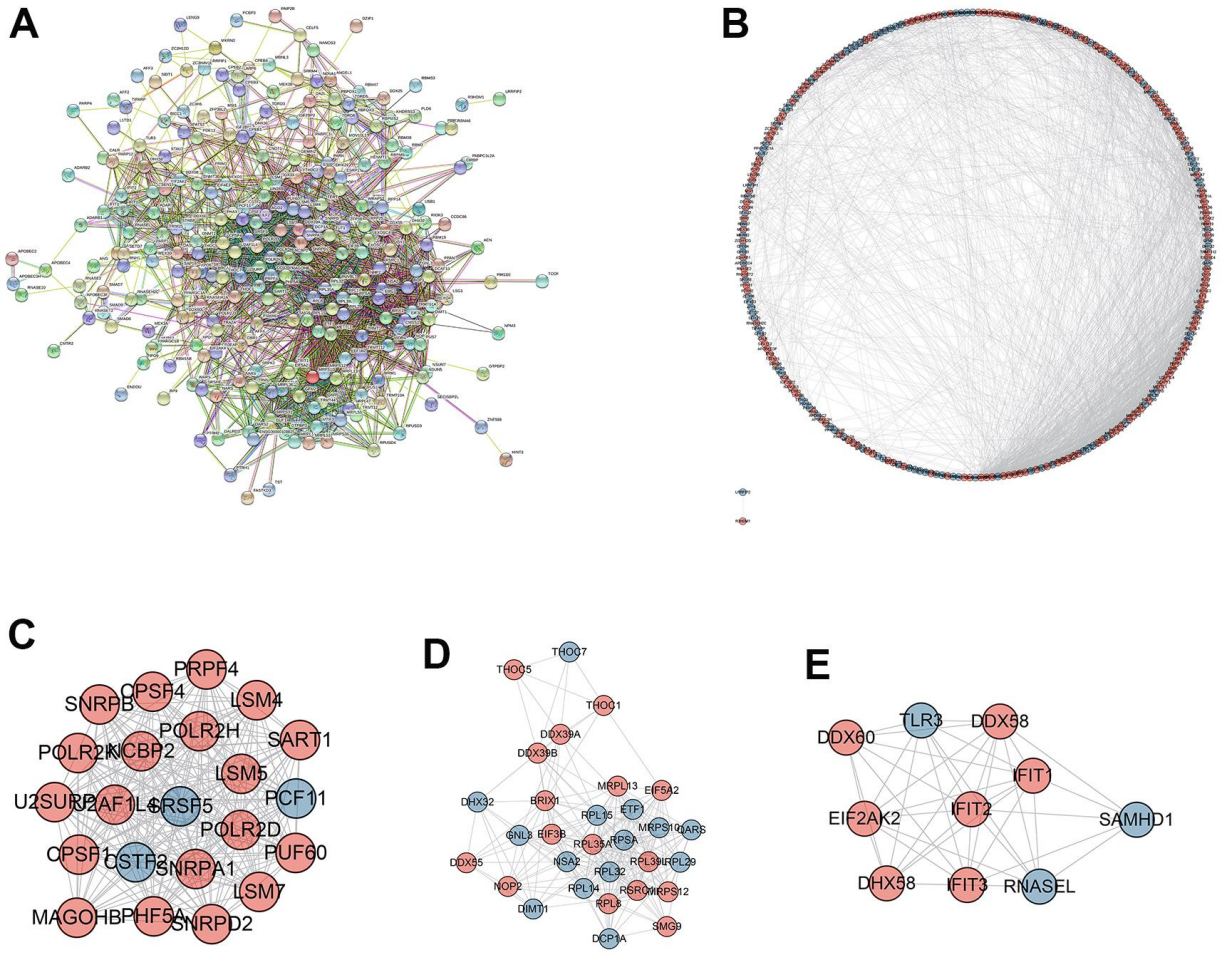
**Protein-protein interaction (PPI) network and module analysis.** (**A**) PPI network of integrated DERBPs by String database; (**B**) Visualized PPI analysis of DERBPs based on Cytoscape; (**C**–**E**) Top three modules from the PPI network.

As shown in Figure 3A, 3B, PPI network was established to further screen the hub DERBPs in LSCC. The most three important modules were screened out and shown in Figure 3C–3E. Pathway enrichment analysis of the three key modules showed that the hub DERBPs were related to RNA activities, such as RNA splicing, RNA transport and RNA binding (Supplementary Table 4).

### Identification of a RBPs-associated prognostic signature

**Figure 4:**
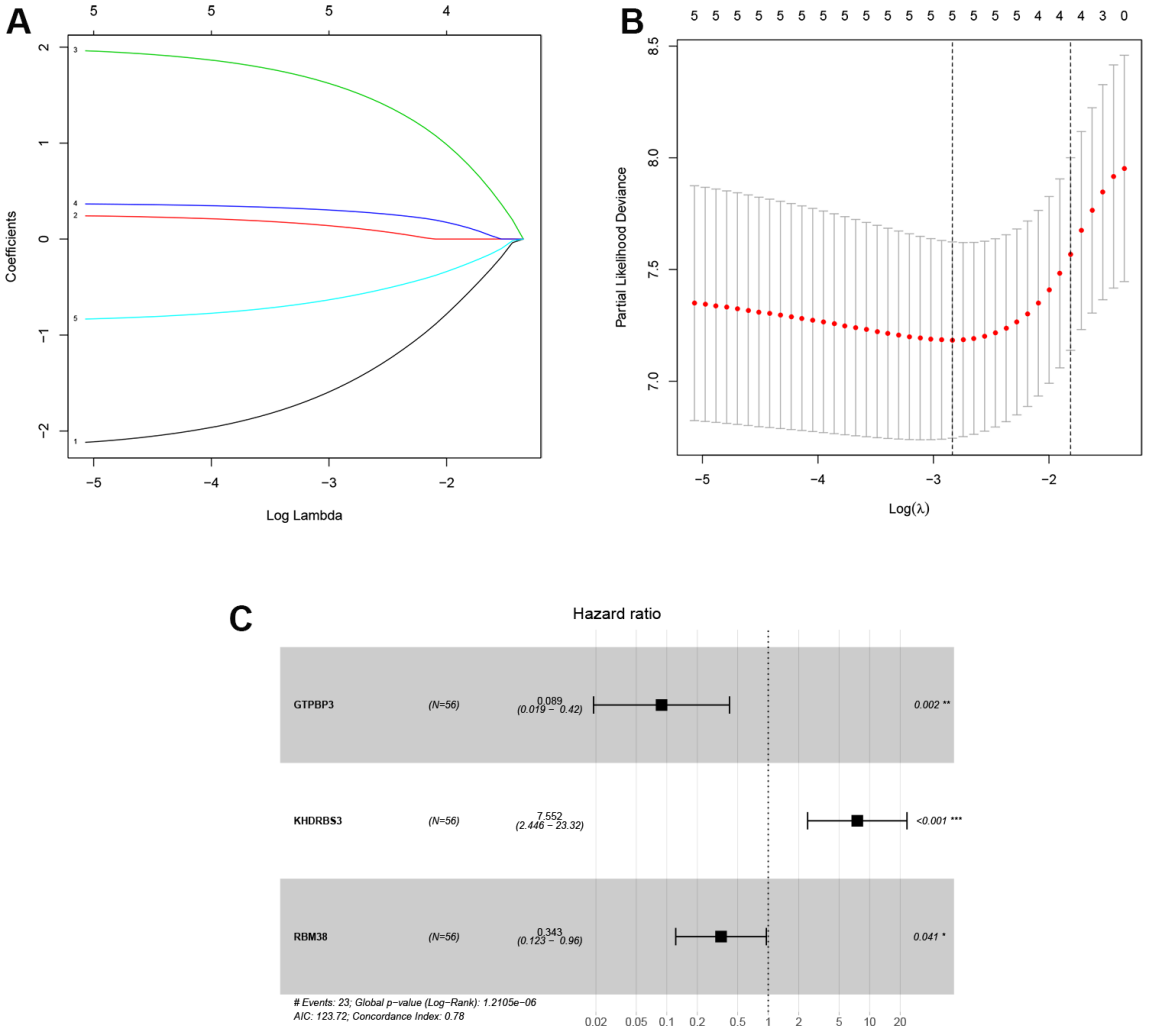
**Identification of prognosis-related DERBPs associated with laryngeal squamous cell carcinoma.** (**A**, **B**) LASSO coefficient profiles of DERBPs selected by univariate Cox regression analysis; (**C**) Forest plot of multivariate Cox regression analysis.

In training cohort, ten RBPs related to prognosis were obtained for the LASSO analysis (Table 2). Next, the LASSO analysis was performed, and the coefficients of five DERBPs are shown in Figure 4. Subsequently, through multivariate Cox regression, three RBPs (GTPBP3, KHDRBS3 and RBM38) were selected to develop the transcriptomic signature for LSCC (Figure 4C and Table 3). The calculation formula of the risk score based on training cohort was described below:

Risk score = (-2.421 * Exp GTPBP3) + (2.022 * Exp KHDRBS3) + (-1.071 * Exp RBM38).

**Table 2:** Univariate Cox regression analysis for prognostic signature.

**id**	**HR**	**HR.95L**	**HR.95H**	***p*value**
KHDRBS3	7.072258353	2.609931172	19.16404492	0.000119996
RBM38	0.216674422	0.078590104	0.597375531	0.003119943
EIF5A2	3.429842961	1.389144386	8.46839454	0.007523681
GTPBP3	0.130350488	0.028933995	0.587241752	0.00797546
CCDC86	4.703911956	1.496876874	14.78196909	0.008038545
TDRD3	7.617503865	1.443551636	40.19694458	0.016732381
CD3EAP	3.877391645	1.156712741	12.99732028	0.028101935
CPEB3	0.028673549	0.000949282	0.86609916	0.041088249
NARS	3.177289338	1.019643854	9.900680028	0.04620559
THOC7	2.769197743	1.003182988	7.644124983	0.04928726

**Table 3:** Multivariate Cox regression analysis for prognostic signature.

**id**	**coef**	**HR**	**HR.95L**	**HR.95H**	***p*value**
GTPBP3	-2.421490619	0.088789168	0.01897852	0.415391518	0.002098438
KHDRBS3	2.021819781	7.552055522	2.445703998	23.31988772	0.000440374
RBM38	-1.070742906	0.342753789	0.122551551	0.958618301	0.041300275

**Figure 5:**
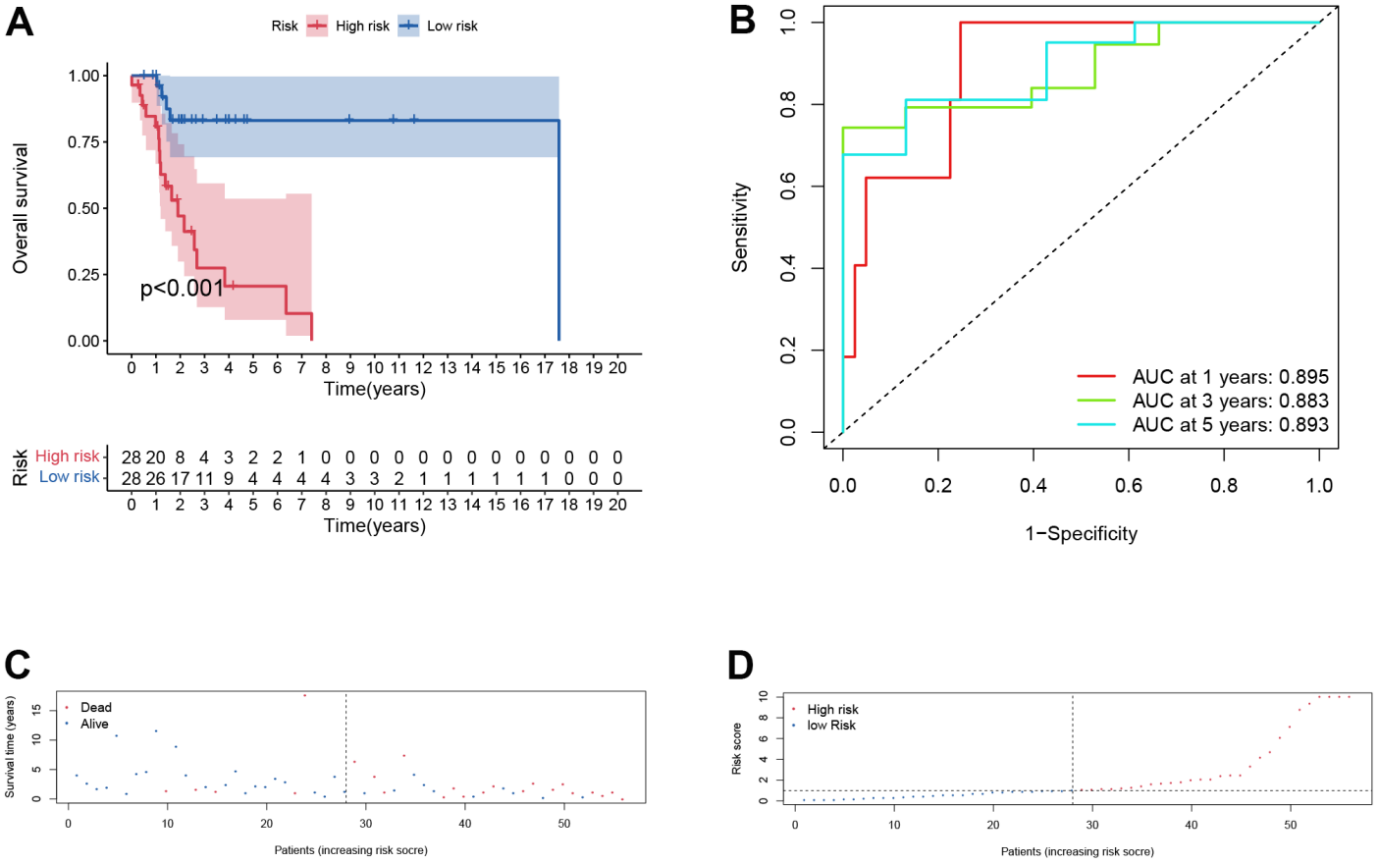
**Prognostic assessment of the transcriptomic signature in training cohort.** (**A**) Kaplan-Meir (KM) survival curves; (**B**) Receiver operating characteristic curves; (**C**, **D**) Risk score distribution and survival status.

In training cohort, there was a worse prognosis for high-risk group compared with low-risk group (*p* < .001, Figure 5A). The one-, three-, and five-years AUC values of the signature was 0.895, 0.883 and 0.89 (Figure 5B), indicating great diagnostic capability of the prognostic signature. The survival status of patients and risk score of the signature consisting of the three DERBPs in the low- and high-risk subgroups were shown in Figure 5C, 5D.

**Figure 6:**
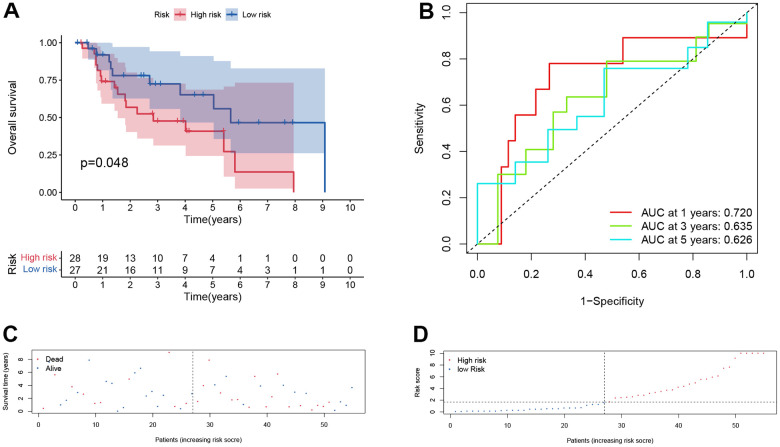
**Prognostic assessment of the transcriptomic signature in training cohort in test cohort.** (**A**) Kaplan-Meir (KM) survival curves; (**B**) Receiver operating characteristic curves; (**C**, **D**) Risk score distribution and survival status.

**Figure 7:**
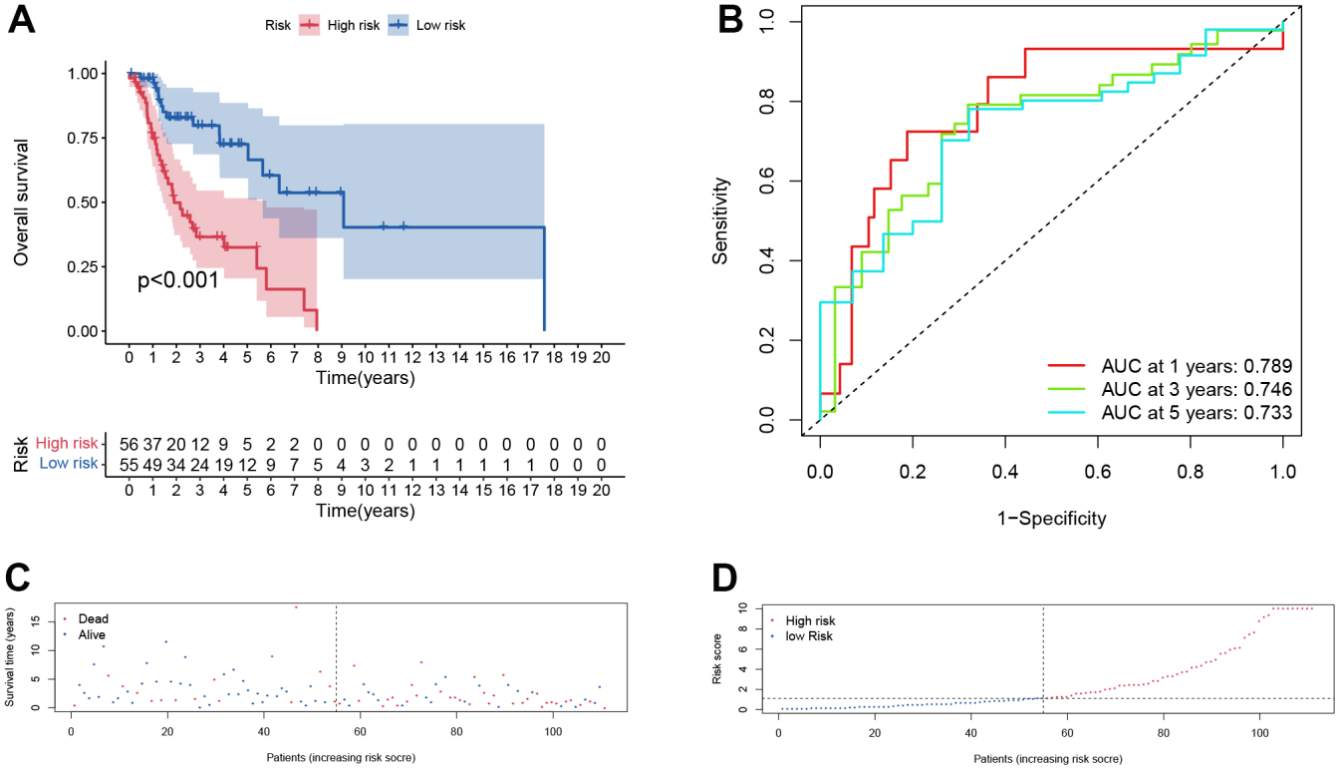
**Prognostic assessment of the transcriptomic signature in TCGA cohort.** (**A**) Kaplan-Meir (KM) survival curves; (**B**) Receiver operating characteristic curves; (**C**, **D**) Risk score distribution and survival status.

Furthermore, the same analysis methods were applied in different cohorts, including test cohort (n=55), entire cohort (n=111), GSE65858 cohort (n=270) and GSE27020 cohort (n=109). In test cohort, patients with a higher risk score also had a worse overall survival than those with a lower risk score (*p* = 0.048, Figure 6A) and entire cohort (*p* < .001, Figure 7A). The Time-dependent AUC values of test cohort and entire cohort were plotted in Figures 6B, 7B. In GSE27020 cohort, patients with higher risk score were more likely to have a worse disease-free survival compared with patients in the lower risk score (*p* = 0.011, Supplementary Figure 1A). The Time-dependent AUC values of GSE27020 cohort were plotted in Supplementary Figure 1B. In GSE65858 cohort (n=270), patients with a higher risk score also had a worse overall survival (*p* = 0.147, Supplementary Figure 2A). The AUC values of the GSE65858 cohort was displayed in Supplementary Figure 2B. As shown in Supplementary Figure 2C, 2D, patients with higher risk score had a higher mortality.

### Immune context of the prognostic signature

To explore the immune characteristics of different subgroups, in TCGA cohort, CIBERSORT (Supplementary Figure 3A) and ssGSEA (Supplementary Figure 3B) algorithms were performed. While only para-inflammation was differentially enriched in the high-risk and low-risk groups, there was no significant difference in immune cell infiltration and TIDE scores between the high-risk and low-risk groups (Supplementary Figure 3C). Additionally, we found that patients in the low-risk group were more sensitive to KIN001-135, MP470, QL-XII-61 and VX-702 (Supplementary Figure 3D–3G).

### Verification by qRT-PCR

**Figure 8:**
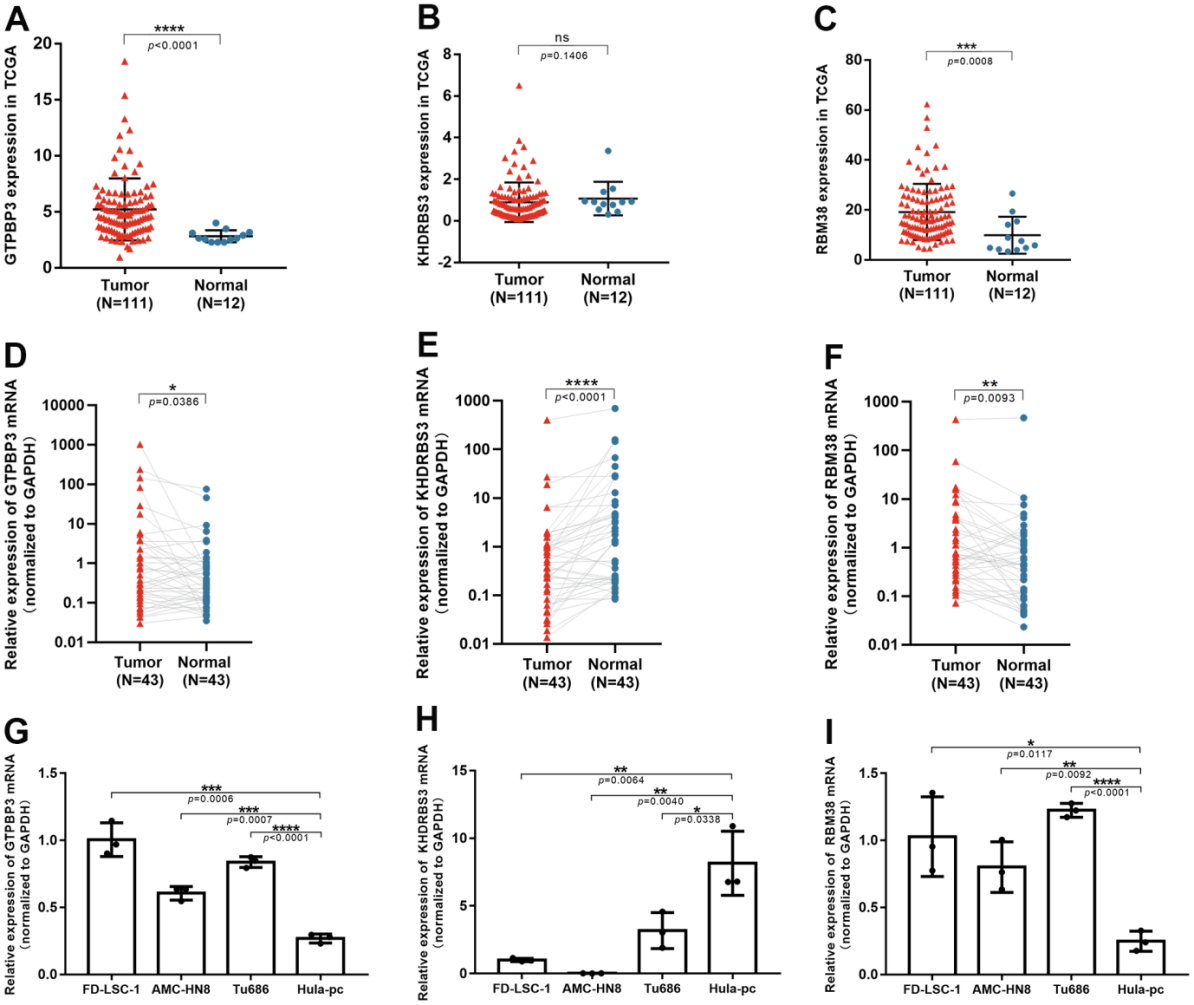
**The validation of three RBPs in tissues and cell lines through qRT-PCR.** (**A**–**C**) Expression of GTPBP3, KHDRBS3 and RBM38 in TCGA database; (**D**–**F**) GTPBP3 and RBM38 presented higher expression in LSCC, compared to their adjacent normal tissue, while KHDRBS3 was the opposite; (**G**–**I**) GTPBP3 and RBM38 presented higher expression in LSCC cell lines, compared to laryngeal epithelial cell line HuLa-PC, while KHDRBS3 was the opposite.

In clinical tissues, higher GTPBP3 and RBM38 expression were found in LSCC tissues, while lower KHDRBS3 expression was found (Figure 8D–8F), which was consistent with the results of differential expression analysis from TCGA (Figure 8A–8C). In cell lines, the same results were noted. (Figure 8G–8I).

### Independent prognosis analysis

**Figure 9:**
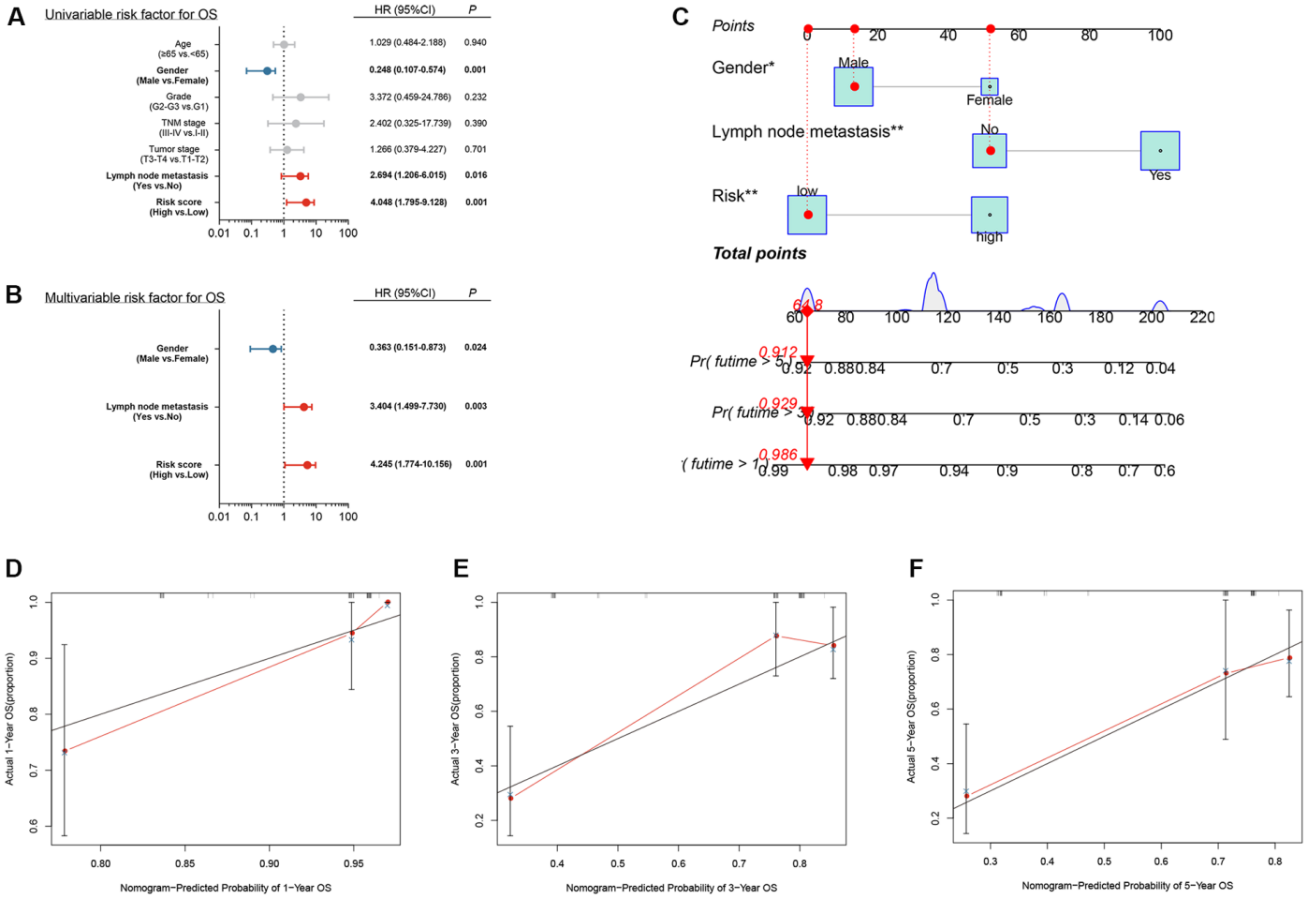
Independent prognostic analysis and construction of a nomogram (**A**) Univariate analysis of clinical factors; (**B**) Multivariate analysis of clinical factors; (**C**) Nomogram for predicting one-, three-, and five-year overall survival of laryngeal squamous cell carcinoma patients based on entire cohort; (**D**–**F**) Calibration plots of the nomogram based on one-, three-, and five-year overall survival. The y-axis represents actual survival, and the x-axis represents nomogram-predicted survival.

Univariate and multivariate Cox regression analysis suggested that gender, lymph node metastasis and risk score were independent prognostic factors of LSCC patients (*p* < .05, Figure 9A, 9B). These results suggested that the risk score calculated by transcriptomic signature was an independent risk factor for patients with LSCC.

### Construction of a nomogram based on the three hub DERBPs

Based on the independent prognosis analysis, a nomogram integrating gender, lymph node metastasis and risk score was developed based on the entire cohort to predict the overall survival of LSCC patients at one, three, and five years after diagnosis (Figure 9C). The calibration curves for predicting one-, three- and five-year overall survival showed that the nomogram-predicted survival rate closely matched the ideal curve (Figure 9D–9F). Our results suggested that, the nomogram we developed according to independent prognosis analysis exhibited great performance for risk stratification of LSCC.

## DISCUSSION

Even with advances in treatment modalities, the prognosis of laryngeal cancer remains poor and the survival rates have not been obviously improved. Hence, it is urgently required to explore molecular mechanisms and identify novel signatures, which could assess the tumor behavior during initial diagnosis in LSCC patients [25]. Nowadays, RNA-sequencing technology was widely applied to development of disease prognostic model [26, 27]. Perfect and effective prognostic features can improve personalized patient management. The large amount of genomic information collected from individual tumor samples has promoted the identification of novel diagnostic, prognostic, or predictive biomarkers [28]. For example, Peng identified a novel gene signature for the prognosis of gastric cancer [29]; Yang found five significant microRNAs related to the prognosis of colorectal cancer [30]; Zhong suggested that six long non-coding RNAs (lncRNAs) might be involved with breast cancer–related biological processes [31]; Zhang identified 214 differentially expressed lncRNAs, and found a novel four-lncRNA signature that could predict the prognosis of cancer [32].

Despite the universal application of prognostic signatures, few signatures were constructed and assessed for laryngeal cancer. To our knowledge, this is the first time that RBPs has been combined with laryngeal cancer. Prognosis-related RBPs were filtered out and a transcriptomic signature was developed according to three RBPs expression (GTPBP3, KHDRBS3, and RBM38).

GTP binding protein 3 (GTPBP3) exists in mitochondria and exerts effects on mitochondria [33]. Past studies suggested that GTPBP3 was associated with some oxidative phosphorylation diseases [33]. As an example, Chen et al. [34] suggested that GTPBP3 was related to mitochondrial tRNA metabolism. Moreover, GTPBP3 is also correlated with primary angle closure glaucoma [35] and non-syndromic hearing loss [36].

KH RNA binding domain containing, signal transduction associated 3 (KHDRBS3) was initially found to be associated with spermatogenesis. Recent research has indicated that KHDRBS3 is strongly involved in tumorigenesis and development. Shi [37] suggested that KHDRBS3 drove circ-0088300 to accelerate the metastasis of gastric carcinoma cells. In colorectal cancer cells, KHDRBS3 was found to promote drug resistance by maintaining stem cell stemness [38]. In this study, lower KHDRBS3 expression was detected in LSCC tissues.

RNA binding motif protein (38RBM38) is located on chromosome 20q13 and is expressed broadly in bone marrow and lymph node [39]. It is worth mentioning that the expression levels of RBM38 are different in different tumor, indicating that the function of RBM38 in tumors is complex. RBM38 acts as a tumor suppressor by reducing c-Myc and enhancing PTEN expression in breast cancer [40]; Observations in most studies suggested that RBM38 promotes cancer [41, 42].

In this study, higher GTPBP3, higher RBM38 and lower KHDRBS3 expression were found in LSCC tissues and cell lines. We developed a transcriptomic signature associated with these prognostic RBPs for laryngeal cancer and verified in internal and external cohorts. Interestingly, based on the results of independent prognostic analysis and the nomogram we constructed, we found that female gender was an independent prognostic factor and females were more likely to suffer from laryngeal cancer. Statistically, laryngeal cancer occurs more commonly in men than in women and the incidence of laryngeal cancer in men is nearly five times that in women [43]. In this retrospective analysis, we found that the prognosis of laryngeal cancer in women was worse than that in men, which could give new hints for our clinical work.

In the previous studies, Duan et al. [44–47] developed a transcriptomic signature based on RBPs in HNSCC. HNSCC contains many regions such as pharynx, larynx, tongue, oral cavity, nasal cavity and paranasal cavity. However, the biological characteristics and overall survival rate of different HNSCC regions are rather divergent. Therefore, it may lead to confusion if many tumor types are enrolled in one study. Based on the principle of precision therapy, we believe studying the function of RBPs in LSCC instead a composition of multiple HNSCC could obtain better results in terms of precision and minimize bias. As far as we know, this was the first attempt to develop a prognostic signature and nomogram based on RBPs for prognostic evaluation in LSCC. In general, the signature showed a higher AUC value than those by other studies [44–47] in predicting 3- and 5-year OS (Supplementary Figure 3H, 3I), indicating a better sensitivity and specificity.

Also, the research we conducted has some shortcomings. The tumor sample size used to construct the transcriptomic signature was small and more LSCC cohorts needed to be collected and included for further analysis. More, molecular biology experiments on GTPBP3, KHDRBS3 and RBM38 need to be performed.

## CONCLUSIONS

In this study, we identified differentially expressed RBPs and screened an innovative three-RBP signature to predict the outcome of LSCC patients for the first time. Results from different independent cohorts showed that this prognostic signature had decent discriminative ability in predicting the prognosis of LSCC patients.

## Supplementary Material

Supplementary Figures

Supplementary Tables 1, 2

Supplementary Table 3

Supplementary Table 4
