# Supplementary material for: Transcriptomic signature associated with RNA-binding proteins for survival stratification of laryngeal cancer
Source: Aging (Albany NY). 2022 Aug 18;14(16):6605–25. doi: 10.18632/aging.204234 (PMC9467394; doi:10.18632/aging.204234)
Supplement: Supplementary Figures [file aging-14-204234-s001.pdf]

SUPPLEMENTARY FIGURES

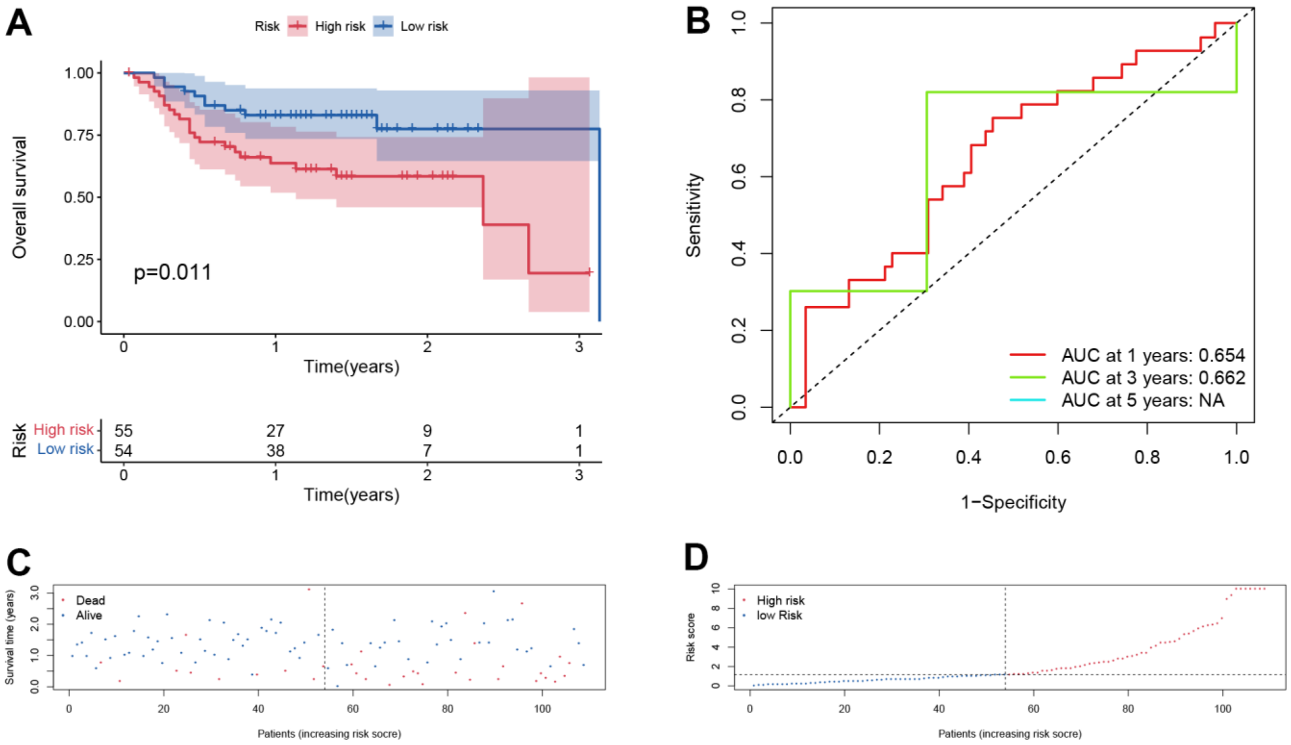

Supplementary Figure 1. Risk score analysis of prognostic model in the GSE27020 cohort. (**A**) Survival curve for the low- and high-risk subgroups; (**B**) Receiver operating characteristic curves for forecasting disease-free survival based on risk score; (**C**, **D**) Risk score distribution and recurrence status.

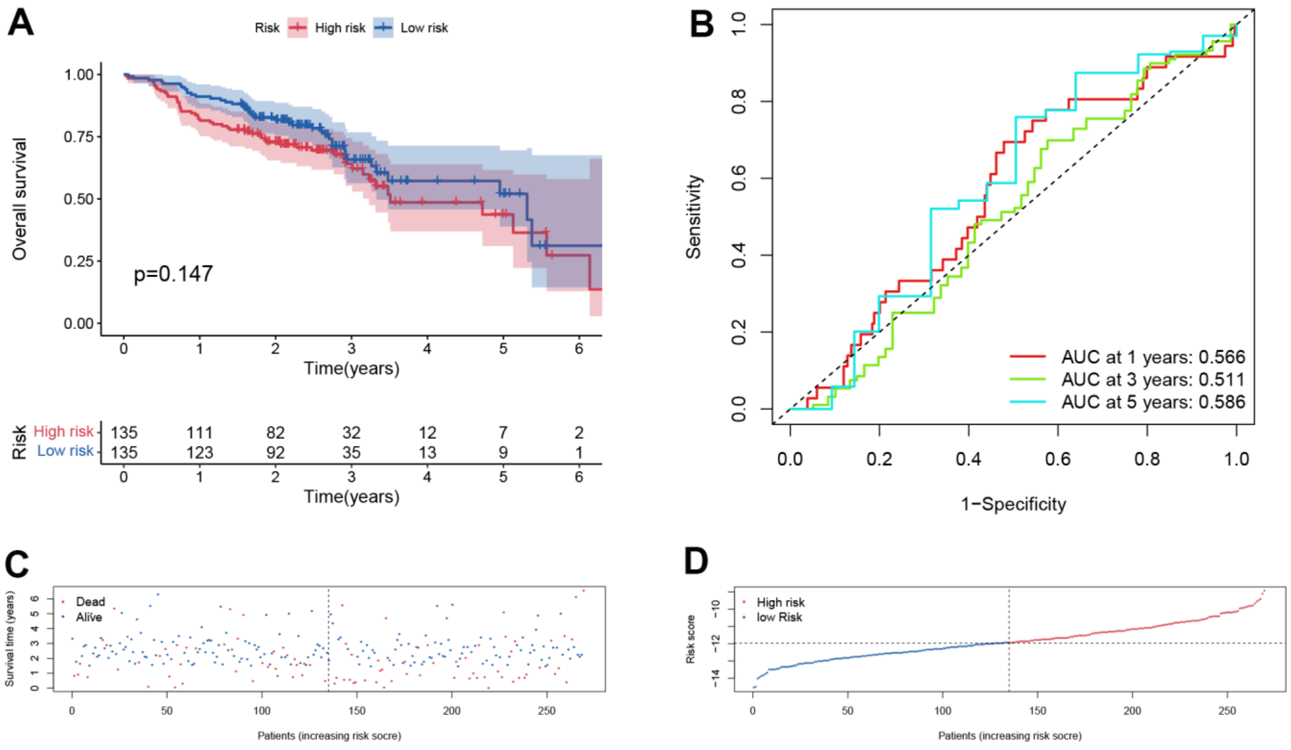

Supplementary Figure 2. Risk score analysis of prognostic model in the GSE65858 cohort. (**A**) Survival curve for the low- and high-risk subgroups; (**B**) Receiver operating characteristic curves for forecasting disease-free survival based on risk score; (**C**, **D**) Risk score distribution and survival status.

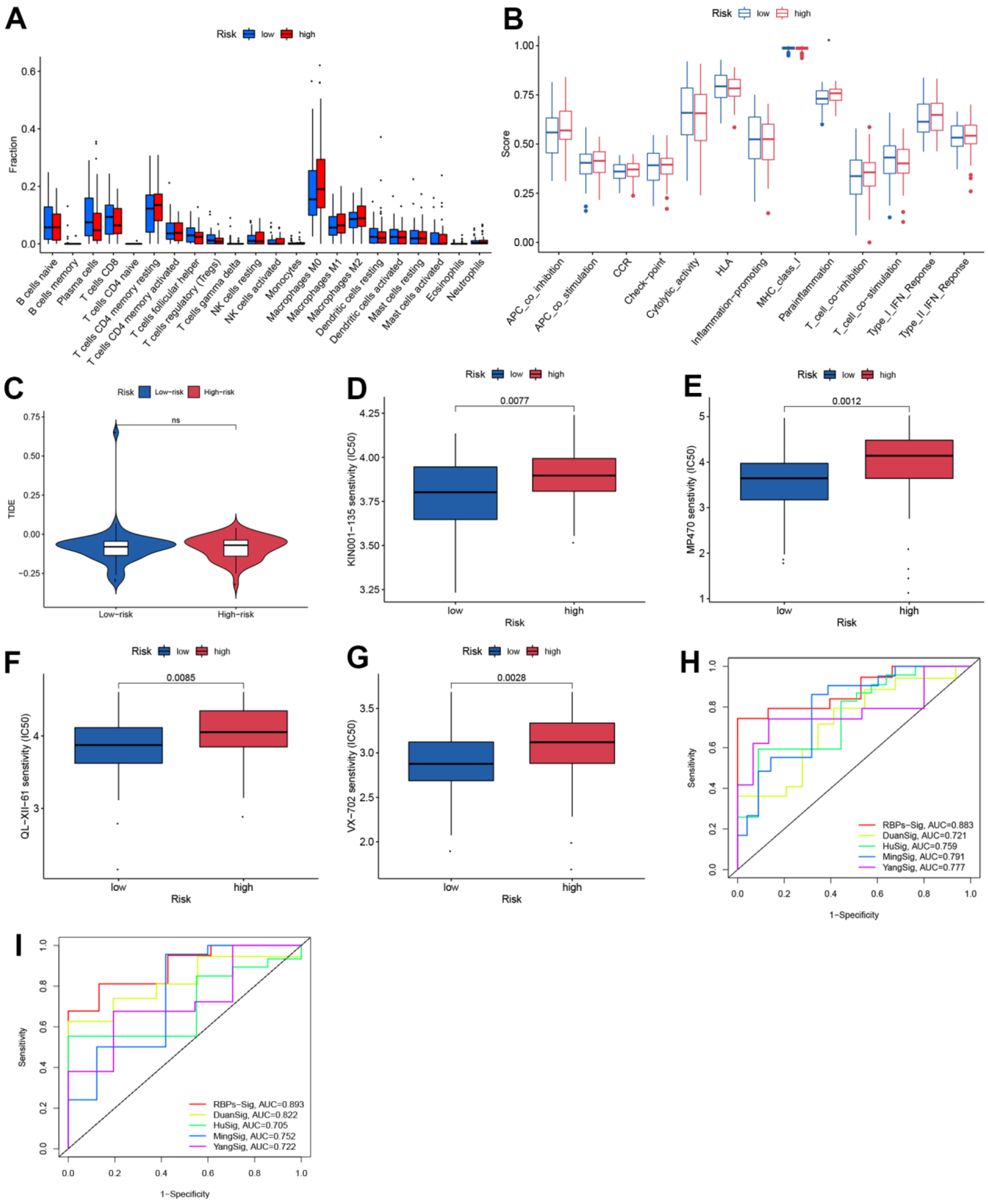

Supplementary Figure 3. Immune context and sensitive drugs between subgroups. (**A**) Immune cell infiltration between subgroups with CIBERSORT algorithm; (**B**) Immune function activity between subgroups with ssGSEA algorithm; (**C**) Immunotherapy response between subgroups with TIDE scores; (**D**) Estimated IC50 values of KIN001-135; (**E**) Estimated IC50 values of MP470; (**F**) Estimated IC50 values of QL-XII-61; (**G**) Estimated IC50 values of VX-702; (**H**) ROC curves of similar methods for predicting three-year OS; (**I**) ROC curves of similar methods for predicting five-year OS.
