# Supplementary material for: Transcriptomic signature associated with RNA-binding proteins for survival stratification of laryngeal cancer
Source: Aging (Albany NY). 2022 Aug 18;14(16):6605–25. doi: 10.18632/aging.204234 (PMC9467394; doi:10.18632/aging.204234)
Supplement: Supplementary Tables 1, 2 [file aging-14-204234-s002.pdf]

# SUPPLEMENTARY TABLES

**Supplementary Table 1. Clinical information of TCGA cohort.**

| Characteristics | Amount(proportion) |
|---|---|
| Age (>60y/ ≤60y) | 47(42.3%)/ 64(57.7%) |
| Gender (Male/ Female) | 91(82%)/ 20(18%) |
| Grade (G1-G2/ G3) | 82(73.9%)/ 29(26.1%) |
| Status (Alive/ Dead) | 61(55%)/ 50(45%) |
| TNM Stage (I-II/III-IV/ Not available) | 12(10.8%)/ 86(77.5%)/ 13(11.7%) |
| T classification (T1-2/ T3-4/ Not available) | 20(18%)/ 78(70.3%)/ 13(11.7%) |
| N classification (N0/ N+/ Not available) | 43(38.7%)/ 55(49.5%)/ 13(11.7%) |

Abbreviation: Grade means pathological grade.

**Supplementary Table 2. The sequences of all primers used in this study.**

| ID | GTPBP3 | KHDRBS3 | RBM38 | GAPDH |
|---|---|---|---|---|
| Forward primer(5'-3') | GCAGGCGAGTTCACCAGAC | TTCCAGTGGTTCGAGGGAAAC | CTGCCGTACCACACTACCG | GATGCCCCCATGTTCGTCAT |
| Reverse primer(5'-3') | TTTCCGCGTGGATAAGGTCC | CTCGTGGTACTACAACTCCAAC | ATGATGGGGTTCGGGTCTTTG | TAAGCAGTTGGTGGTGCAGG |
