# Supplementary material for: Transcriptomic signature associated with RNA-binding proteins for survival stratification of laryngeal cancer
Source: Aging (Albany NY). 2022 Aug 18;14(16):6605–25. doi: 10.18632/aging.204234 (PMC9467394; doi:10.18632/aging.204234)
Supplement: Supplementary Table 4 [file aging-14-204234-s004.pdf]

Supplementary Table 4. Gene ontology and Kyoto Encyclopedia of Genes and Genomes pathway analysis of the differentially expressed RNA-binding proteins in the key modules.

	Description	*p* value	*p*.adjust
**subNet1**			
	ribosome biogenesis	1.23604819956605E-53	2.47726683998237E-51
	rRNA processing	1.80164861089627E-53	2.47726683998237E-51
**BP**	ncRNA processing	3.49783767023767E-52	3.20635119771786E-50
	rRNA metabolic process	4.58359810633518E-51	3.15122369810544E-49
	maturation of SSU-rRNA	5.99452455159044E-12	3.29698850337474E-10
	preribosome	6.34171978636655E-33	2.09276752950096E-31
	small-subunit processome	1.04130348165355E-23	1.71815074472837E-22
**CC**	nucleolar part	3.22657076243753E-20	3.54922783868128E-19
	90S preribosome	4.99058966692517E-13	4.11723647521327E-12
	fibrillar center	1.48178141267309E-11	9.77975732364241E-11
	snoRNA binding	2.6734745200065E-13	1.73775843800423E-11
	catalytic activity, acting on RNA	1.47039551774291E-10	4.77878543266446E-09
**MF**	RNA helicase activity	9.73584405162519E-10	2.10943287785213E-08
	helicase activity	1.67118431651836E-07	2.71567451434234E-06
	RNA methyltransferase activity	6.73210692872447E-07	8.75173900734181E-06
**KEGG**	Ribosome biogenesis in eukaryotes	4.87604513359848E-29	9.75209026719696E-29
**subNet2**			
	mitochondrial translational elongation	3.19077661737608E-24	1.9770698154501E-23
	mitochondrial translational termination	3.59467239172745E-24	1.9770698154501E-23
**BP**	translational termination	1.84511620668653E-23	6.76542609118395E-23
	translational elongation	2.3853363994251E-22	6.12462051769803E-22
	mitochondrial translation	2.78391841713547E-22	6.12462051769803E-22
	organellar ribosome	1.6386550585062E-24	1.31092404680496E-23
	mitochondrial ribosome	1.6386550585062E-24	1.31092404680496E-23
**CC**	ribosomal subunit	5.43877874796791E-21	2.90068199891622E-20
	mitochondrial protein complex	1.44572305331084E-19	5.78289221324337E-19
	ribosome	2.11626701607867E-19	6.77205445145175E-19
	structural constituent of ribosome	3.00137078934191E-20	1.20054831573676E-19
	small ribosomal subunit rRNA binding	2.13874983828877E-08	4.27749967657753E-08
**MF**	rRNA binding	5.06850011824096E-06	6.75800015765461E-06
	oxidoreductase activity, acting on the aldehyde or oxo group of donors	0.024039989892977	0.024039989892977
**KEGG**	Ribosome	2.77702116923204E-09	2.77702116923204E-09
**subNet3**			
	piRNA metabolic process	9.14280943940186E-12	1.84684750675917E-09
	RNA splicing, via transesterification reactions with bulged adenosine as nucleophile	1.26242187179221E-10	6.83716002246461E-09
**BP**	mRNA splicing, via spliceosome	1.26242187179221E-10	6.83716002246461E-09
	RNA splicing, via transesterification reactions	1.35389307375537E-10	6.83716002246461E-09
	RNA splicing	8.29844787638623E-10	3.35257294206004E-08
	P granule	2.5559092533254E-14	5.96378825775926E-13
	pole plasm	2.5559092533254E-14	5.96378825775926E-13
**CC**	germ plasm	2.5559092533254E-14	5.96378825775926E-13
	cytoplasmic ribonucleoprotein granule	2.82428926655342E-11	4.94250621646848E-10
	ribonucleoprotein granule	4.23382682510392E-11	5.92735755514549E-10
**MF**	catalytic activity, acting on RNA	0.0000434647392351823	0.00226016644022948

	ribonucleoprotein complex binding	0.000368208665236822	0.00957342529615737
	helicase activity	0.00066694155273121	0.011560320247341
	snRNA binding	0.00114700790836894	0.0140337338176663
	translation initiation factor activity	0.00134939748246791	0.0140337338176663
**KEGG**	Spliceosome	3.82425188294518E-06	0.0000229455112976711
	RNA transport	0.0097535710768834	0.0292607132306502
